# Pharmacokinetics of oral and intravenous melatonin in healthy volunteers

**DOI:** 10.1186/s40360-016-0052-2

**Published:** 2016-02-19

**Authors:** Lars P. H. Andersen, Mads U. Werner, Mette M. Rosenkilde, Nathja G. Harpsøe, Hanne Fuglsang, Jacob Rosenberg, Ismail Gögenur

**Affiliations:** Department of Surgery D, Herlev Hospital, University of Copenhagen, Herlev, DK-2730 Denmark; Multidisciplinary Pain Center 7612, Neuroscience Center, Rigshospitalet, Copenhagen, DK-2100 Denmark; Department of Neuroscience and Pharmacology, University of Copenhagen, Copenhagen, DK-2100 Denmark; Department of Surgery, Roskilde and Køge Hospital, University of Copenhagen, Roskilde, DK-4000 Denmark

**Keywords:** Bioavailability, Intravenous, Melatonin, Oral, Pharmacokinetic

## Abstract

**Background:**

The aim was to investigate the pharmacokinetics of oral and iv melatonin in healthy volunteers.

**Methods:**

The study was performed as a cohort crossover study. The volunteers received either 10 mg oral melatonin or 10 mg intravenous melatonin on two separate study days. Blood samples were collected at different time points following oral administration and short iv infusion, respectively. Plasma melatonin concentrations were determined by RIA technique. Pharmacokinetic analyses were performed by “the method of residuals” and compartmental analysis. The pharmacokinetic variables: *k*_a_, *t*_1/2 absorption_, *t*_max_, *C*_max_, *t*_1/2 elimination,_*AUC*_0-∞_, and bioavailability were determined for oral melatonin. *C*_max_, *t*_1/2 elimination_, *V*_d_, *CL* and *AUC*_0-∞_ were determined for intravenous melatonin.

**Results:**

Twelve male volunteers completed the study. Baseline melatonin plasma levels did not differ significantly between the study days (*P* = 0.067). Mean (SD) *t*_1/2 absorption_ of oral melatonin was 6.0 (3.1) min. Mean *t*_max_ was 40.8 (17.8) min with a median (IQR) *C*_max_ of 3550.5 (2500.5–8057.5) pg ml^-1^. Mean *t*_1/2 elimination_ was 53.7 (7.0) min. Median absolute bioavailability was 2.5 (1.7–4.7) %. Median *C*_max_ after short iv infusion of melatonin was 389,875.0 (174,775.0–440,362.5) pg ml^-1^. Mean *t*_1/2 elimination_ was 39.4 (3.6) min, mean *V*_d_ 1.2 (0.6) l kg^-1^ and mean *CL* 0.0218 (0.0102) l min^-1^ kg^-1^.

**Conclusions:**

This cohort crossover study estimated pharmacokinetics of oral and iv melatonin, respectively in healthy volunteers. Bioavailability of oral melatonin was only 3 %.

**Trial registration:**

Eudra-CT number: 2013-000205-23 (initial registration 27.03.2013).

Clinicaltrials.gov Identifier: NCT01923974 (initial registration 08.08.2013).

## Background

Exogenous melatonin is being increasingly employed as treatment for various medical and surgical diseases [1, 2]. Furthermore, a recent study, administering intravenous (iv) melatonin has documented reduced cardiac morbidity and markers of myocardial ischemia following elective abdominal aortic aneurism repair [3]. Despite its widespread clinical use, the pharmacokinetic properties of exogenous melatonin still need to be established further [4]. A limited number of experimental studies in healthy volunteers have performed *direct* comparisons of the pharmacokinetics of oral and iv melatonin [5, 6]. The studies differed in number of investigated subjects, dosages, methods and pharmacokinetic analyses [5, 6]. Accordingly, the pharmacokinetic variables varied extensively between the studies [5, 6]. In order to achieve an optimized clinical efficacy of melatonin, further investigation of the pharmacokinetics is clearly needed.

The aim of the study was to investigate the pharmacokinetics of oral and iv melatonin in a cohort of healthy volunteers.

## Methods

Approvals were obtained from the Capital Region’s Committee on Health Research Ethics (Protocol number: H-4-2013-013), the Danish Health and Medicines Authority (Eudra-CT number: 2013-000205-23, Clinicaltrials.gov Identifier: NCT01923974) and the Danish Data Protection Agency (Journal number: HEH-2013-008, nr: 02095) prior to inclusion of volunteers. Informed written- and verbal consent were provided by all volunteers. The blood samples relating to iv melatonin were obtained from a primary trial investigating analgesic and anti-hyperalgesic effects of melatonin (Eudra-CT number: 2013-000205-23, Clinicaltrials.gov Identifier: NCT01923974). Data presented in the present paper have not been published previously.

The study was performed as a cohort crossover study. The volunteers received either 10 mg of oral melatonin or 10 mg of iv melatonin on two separate study days. Each study session was performed from 08:00 a.m. to 04:00 p.m. The study days were separated by 3 to 9 months. Inclusion criteria were age 20–40 years and male gender. Volunteers were excluded, if they did not understand written- or spoken Danish, suffered from serious physical or mental illness, were diagnosed with a sleep disorder, worked night shifts, received daily analgesics, had participated in other clinical trials within one month from study inclusion or suffered from skin abnormalities on the lower extremities (due to the test paradigm applied in the primary trial).

Oral melatonin consisted of one gelatine capsule containing 10 mg melatonin. Volunteers were allowed 5 cl of tap water to facilitate oral intake. Volunteers were instructed to adhere to preoperative fasting guidelines before the study session with oral melatonin (liquids: minimum fasting period = 2 h; meals: minimum fasting period = 6 h) [7]. Volunteers were encouraged to drink and eat 2 h following oral melatonin administration. Intravenous melatonin consisted of a 25 ml ethanol/saline solution (2 ml 99.9 % ethanol/23 ml 0.9 % saline) containing 10 mg of melatonin (Helsinn Chemicals SA, Biasca, Switzerland). Intravenous melatonin was administered as short iv infusion in the *left* antecubital vein (2.5 ml min^-1^; 10 min duration).

Blood samples were collected from a peripheral venous catheter inserted in the *right* antecubital vein at specified time points during each study session. The time points differed between oral and iv melatonin. Oral melatonin: at baseline (before medication = endogenous melatonin production), and 0, 10, 20, 30, 40, 50, 60, 70, 80, 90, 100, 110, 120, 180, 240, 300, 360 and 420 min after oral administration. Intravenous melatonin: at baseline, and 0, 60, 120, 180, 240, 300, 360 and 420 min after short iv infusion. Before each blood sample was collected, a 3 ml volume of blood (residual volume) was drawn from the peripheral venous catheter and discarded. Fractionation of blood samples was performed at 5000 r.p.m. for 5 min, and plasma samples were stored at -80 °C. Quantitative determination of plasma melatonin concentrations was performed by radioimmunoassay (RIA)-technique (Melatonin Direct RIA, DIAsource, Louvaine-La-Neuve, Belgium). Precision of the RIA kit: intra-assay coefficient of variation (CV) = 9.8–13.4 %, inter-assay CV = 8.0–13.3 %. The limit of detection was 2.3 pg ml^-1^. Linearity of the kit ranged between 8.5–529.0 pg ml^-1^. If plasma concentrations exceeded detection range of the kit, plasma samples were diluted according to manufacturer’s guidelines. All plasma samples were analysed in duplicate, and the mean value was reported.

### Statistical and pharmacokinetic analyses

Normality of data was assessed by visual inspection of residual plots and histograms. Parametric or non-parametric tests were applied according to the distribution of data. Correspondingly, data are presented as mean (SD) or median (IQR), unless stated otherwise. A *P*-value < 0.05 is considered statistically significant. Data were analysed using IBM SPSS Statistics for Windows version 22.0 (IBM Corp., Armonk, NY, USA) and Graph Pad Prism version 6.0 (Graph Pad Software Inc., La Jolla, CA, USA).

The baseline melatonin plasma concentrations of each study day were compared using a paired sample T-test. Baseline levels were *not* subtracted from post-treatment (*oral* or *iv*) levels.

Pharmacokinetic analyses of *oral* and *iv* melatonin were performed separately.

#### Oral melatonin

Time to maximal concentrations (*t*_max_) and maximal plasma concentrations (*C*_max_) were assessed directly at the relevant time points. The pharmacokinetic variables: absorption constant (*k*_a_), absorption half-life (*t*_1/2 absorption_), elimination rate constant (*k*_e_) and elimination half-life (*t*_1/2 elimination_) were estimated by “the method of residuals” [8]. Areas-under-the-curve (AUC) of plasma concentrations were calculated by applying the trapezoidal rule [9]. *AUC*_0-∞_ was estimated as *AUC*_0-420 min_ + (*C*_420 min_/*k*_e_). Bioavailability was calculated as (AUC_0-∞ oral_ / AUC_0-∞ IV_) x 100.

#### Intravenous melatonin

*C*_max_ was assessed directly at the time point, 0 min after short iv infusion. Pharmacokinetic variables were calculated by compartmental analysis [10]. The pharmacokinetic variables: *t*_1/2 elimination_, volume of distribution (*V*_d_) and clearance (*CL*) were estimated from individual linear regression lines of log-transformed (natural logarithm) plasma concentrations. Following standard equations were applied: *t*_1/2 elimination_ = ln (2) / *k*_e_, *V*_d_ = dose / *C*_0 min_, *CL* = *k*_e_ x *V*_d_. “Goodness of fit” of the individual linear regression lines was assessed by the coefficient of determination, R^2^. *AUC*_0-∞ IV_ was estimated, as described above.

## Results

Twelve male volunteers were included and completed the study. Mean age and body mass index (BMI) were 27.1 (5.2) years and 23.2 (2.7) kg m^-2^, respectively. Baseline melatonin plasma concentrations did not differ significantly between the study days (before oral melatonin = 27.3 (13.5) pg ml^-1^; before intravenous melatonin = 18.3 (12.3) pg ml^-1^) (*P* = 0.067).

The pharmacokinetic variables of oral and iv melatonin are presented in Tables 1 and 2.

**Table 1:** Pharmacokinetic variables of 10 mg of oral melatonin

*t*_1/2 absorption_ min	*t*_max_ min	*C*_max_ pg mL^-1^	*t*_1/2 elimination_ min	*AUC*_0-∞ oral _ pg ml^-1^ min	*f* %
6.0 (3.1)	40.8 (17.8)	3550.5 (2500.5–8057.5)	53.7 (7.0)	281,538.3 (232,696.1–546,285.4)	2.5 (1.7–4.7)

Absorption half-life, time to maximal concentration and elimination half-life data are presented as mean (SD). Maximal concentration, area-under-the-curve and bioavailability data are presented as median (IQR)

Absorption half-life, *t*_1/2 absorption_; time to maximal concentration, *t*_max_; maximal plasma concentration, *C*_max_; elimination half-life, *t*_1/2 elimination_; area-under-the-curve, *AUC*; bioavailability, *f*

**Table 2:** Pharmacokinetic variables of 10 mg of iv melatonin

*C*_max_ pg ml^-1^	*t*_1/2 elimination_ min	*V*_d_ l kg^-1^	*CL* l min^-1^ kg^-1^	R^2^	*AUC*_0-∞ IV_ pg ml^-1^ min
389,875.0 (174,775.0–440,362.5)	39.4 (3.6)	1.2 (0.6)	0.0218 (0.0102)	0.96 (0.93–0.97)	14,179,767.6 (7,063,347.4–18,964,804.0)

Maximal concentration, coefficient of determination and area-under-the-curve data are presented as median (IQR). Elimination half-life, volume of distribution and clearance data are presented as mean (SD)

Maximal plasma concentration, *C*_max_; elimination half-life, *t*_1/2 elimination_; volume of distribution, *V*_d_; clearance, *CL*; coefficient of determination, R^2^; area-under-the-curve, *AUC*

### Oral melatonin

**Fig. 1:**
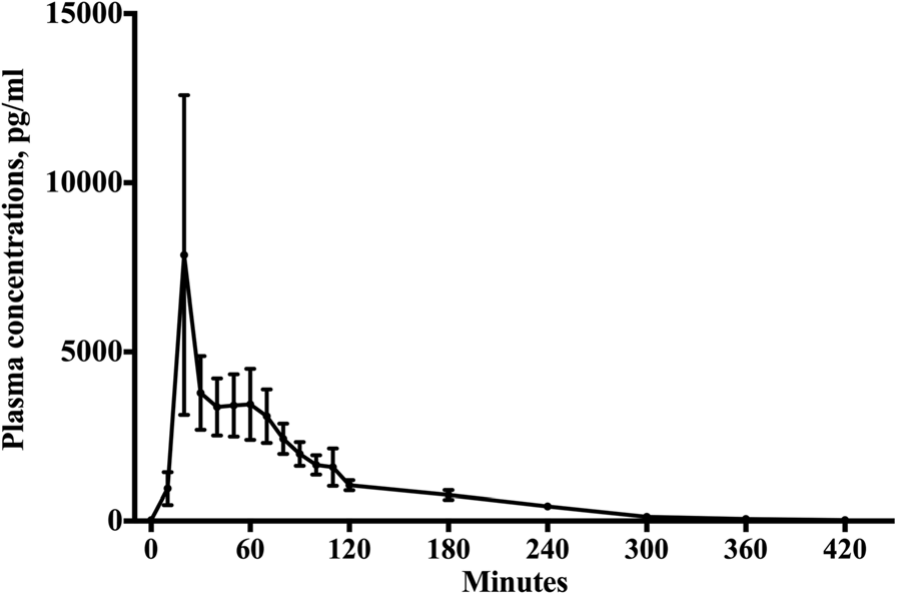
Pharmacokinetic profile of 10 mg of oral melatonin. The dots represent mean values. Whiskers represent SEM

Oral melatonin demonstrated first-order absorption and elimination kinetics. Mean *k*_a_ was 0.2 (0.1) min^-1^, and mean *t*_1/2 absorption_ of oral melatonin was 6.0 (3.1) min (Fig. 1). Mean *t*_max_ was 40.8 (17.8) min with a median (IQR) *C*_max_ of 3550.5 (2500.5–8057.5) pg ml^-1^. Mean *t*_1/2 elimination_ was 53.7 (7.0) min, *AUC*_0-∞ oral_ 281,538.3 (232,696.1–546,285.4) pg ml^-1^ min and median absolute bioavailability was 2.5 (1.7–4.7) %.

### Intravenous melatonin

**Fig. 2:**
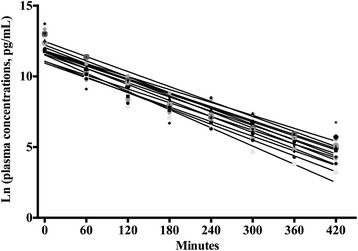
Individual pharmacokinetic profiles of 10 mg of iv melatonin

The pharmacokinetic profiles of iv melatonin demonstrated first-order elimination kinetics (Fig. 2). Median *C*_max_ after iv bolus injection of 10 mg melatonin was 389,875.0 (174,775.0–440,362.5) pg ml^-1^. Mean *t*_1/2 elimination_ was 39.4 (3.6) min, mean *V*_d_ 1.2 (0.6) l kg^-1^ and mean *CL* 0.0218 (0.0102) l min^-1^ kg^-1^. Median R^2^ was 0.96 (0.93–0.97). Median *AUC*_0-∞ IV_ vas 14,179,767.6 (7,063,347.4–18,964,804.0) pg ml^-1^ min.

## Discussion

This cohort crossover study demonstrated a *t*_max_ of 41 min following oral administration. *C*_max_ and *AUC* varied extensively between volunteers in both administration routes. Elimination half-lives were 54 min and 39 min, respectively. Bioavailability of oral melatonin was only 3 %, but demonstrated substantial inter-individual differences.

### Oral melatonin

Oral melatonin was absorbed by first-order kinetics, which has previously been demonstrated in doses up to 80 mg [8]. The short *t*_1/2 absorption_ of 6 min, corroborate studies, applying similar oral drug formulations [8]. Accordingly, our *t*_max_ value of 41 min is in agreement with other studies, documenting values ranging from 30 to 60 min [6, 11]. Oral administration of exogenous melatonin, approximately 45 min before intended onset of clinical effects therefore seems reasonable, assuming that clinical efficacy coincides with *t*_max_ values [12]. Oral administration was associated with extremely variable *C*_max_ and *AUC*_0-∞ oral_ values, which has been described previously [5]. The inter-individual variations are apparently caused by differences in absorption, distribution, metabolism or excretion of the drug, but the exact causes and clinical implications remain unestablished so far [5]. Previous studies demonstrate *t*_1/2 elimination_ values ranging from 46 to 65 min in oral doses from 0.5 to 6 mg [5, 6, 11], which correlates with our findings of 54 min. Our data demonstrated a very low absolute bioavailability of 3 %, albeit with a substantial inter-individual variability. Previous experimental studies have documented higher values ranging between 9 and 33 %, although with comparable inter-individual variability [5, 6, 12]. It is well established in both animal- and human studies that the low bioavailability results from an extensive hepatic first pass metabolism [5]. Similarly, it is also clear that these findings may mandate future dose regulations between different administration routes. However, a general lack of experimental- and clinical studies correlating melatonin plasma concentration levels and clinical effects still remains, and further knowledge is needed, preferably by *in-depth* pharmacokinetic-pharmacodynamic modelling.

### Intravenous melatonin

Previous studies investigating iv administration of melatonin have also demonstrated first-order eliminations kinetics [10], as observed in our study. As with oral melatonin, iv administrations displayed extensive variations in *C*_max_ and *AUC*_0-∞ IV_ values, which is in accordance with previous studies [6]. Other studies also documented *t*_1/2 elimination_ values ranging between 28 and 60 min in iv doses from 0.005 mg to 2 mg [6, 10, 13], which corresponds to the 39 min, demonstrated in the present study. Several studies confirm that elimination rates of iv melatonin (and oral melatonin) are not related to the administered dose. Similarly, previous studies document *CL* values of 0.013 l min^-1^ kg^-1^ (weight-corrected) [13] and 0.027 l min^-1^ kg^-1^ [10], which correspond well to our findings of 0.022 l min^-1^ kg^-1^. Cavallo and colleagues also documented a *V*_D_ of 1.8 l kg^-1^ [10], which is comparable with a value of 1.2 l kg^-1^, demonstrated in our study.

### Strengths

Our study is the first to perform direct comparisons of pharmacokinetics of oral and iv melatonin in doses routinely administered perioperatively (*approximately 10 mg*) [2]. The study was performed as a crossover study to reduce the effect of the inter-individual variability on pharmacokinetic data. Our experimental setup included multiple blood samples for a detailed description of both absorption and elimination phases in both administration routes. Our study also included standard pharmacokinetic methods, such as “the method of residuals” and compartmental analysis [8, 10]. In addition, we chose to include the coefficient of determination (R^2^) to document the “goodness of fit” of the individual linear regression lines in the compartmental analysis. Our data demonstrated a R^2^ value of 0.96, indicating a high degree of “fit” of the first-order pharmacokinetic model, and, hence, a considerable accuracy of the derived pharmacokinetic variables.

### Limitations

*First*, this study only included healthy male volunteers in an experimental setup. Hence, a potential gender difference in pharmacokinetic variables may exist. Furthermore, previous experimental studies indicate that the pharmacokinetics of melatonin is affected by age [10] and external factors, such as caffeine intake [14] cigarette smoking [15] and the use of oral contraceptives [16]. Also, a low number of clinical studies have demonstrated altered pharmacokinetic variables of melatonin [17–19] in e.g. critically ill patients [17, 18]. Interestingly, most other patient groups, e.g. surgical patients, still remain to be investigated. Comorbidity and drug interactions may change the pharmacokinetics of melatonin, potentially altering clinical efficacy of the drug [20].

*Second*, oral and iv study sessions were separated by 3 to 9 months for each volunteer. These time periods may theoretically have affected the comparability of individual pharmacokinetic variables, despite the crossover design. It, however, seems unlikely, as all volunteers were healthy young males in stable physical conditions.

*Third*, the very low bioavailability of oral melatonin documented in our study may indicate a deficient absorption of the drug in our setup. The volunteers were allowed 5 cl of tap water to facilitate intake of oral melatonin. Hence, it can be discussed, if the restricted liquid volume, despite saliva and gastric/intestinal fluid secretions was sufficient to dissolve and present the ingested melatonin to the small intestine, where absorption is mainly achieved. We, however, chose this amount of water to standardize the experimental conditions and to imitate a clinical premedication scenario [2, 7]. Also, we administered an easily absorbable gelatine capsule in order to optimize dissolution of the drug. Finally, comparable *t*_max_ values between the volunteers were demonstrated, suggesting that an impeded absorption is rather unlikely.

## Conclusions

This crossover cohort study investigated the pharmacokinetics of oral and intravenous melatonin in healthy male volunteers. Oral melatonin was rapidly absorbed, and T_max_ was achieved after 41 min. *C*_max_ and *AUC* varied extensively between volunteers. Elimination half-lives following oral and intravenous melatonin administration was 54 min and 39 min, respectively. The bioavailability of oral melatonin was only 3 %, but a considerable variability between the volunteers was noted.
